# The influence of NQO2 on the dysfunctional autophagy and oxidative stress induced in the hippocampus of rats and in SH‐SY5Y cells by fluoride

**DOI:** 10.1111/cns.14090

**Published:** 2023-01-17

**Authors:** Long‐Yan Ran, Jie Xiang, Xiao‐Xiao Zeng, Wen‐Wen He, Yang‐Ting Dong, Wen‐Feng Yu, Xiao‐Lan Qi, Yan Xiao, Kun Cao, Jian Zou, Zhi‐Zhong Guan

**Affiliations:** ^1^ Department of Pathology at the Affiliated Hospital of Guizhou Medical University Key Laboratory of Endemic and Ethnic Diseases (Guizhou Medical University) of the Ministry of Education Guiyang China; ^2^ Department of Medical Science and Technology Guiyang Healthcare Vocational University Guiyang China; ^3^ Key Laboratory of Endemic and Ethnic Diseases (Guizhou Medical University) of the Ministry of Education and Provincial Key Laboratory of Medical Molecular Biology Guiyang China; ^4^ Department of Hepatobiliary Surgery Affiliated Hospital to Guizhou Medical University Guiyang China

**Keywords:** autophagy, brains, experimental fluorosis, NQO2, oxidative stress

## Abstract

**Introduction:**

For investigating the mechanism of brain injury caused by chronic fluorosis, this study was designed to determine whether NRH:quinone oxidoreductase 2 (NQO2) can influence autophagic disruption and oxidative stress induced in the central nervous system exposed to a high level of fluoride.

**Methods:**

Sprague–Dawley rats drank tap water containing different concentrations of fluoride for 3 or 6 months. SH‐SY5Y cells were either transfected with NQO2 RNA interference or treated with NQO2 inhibitor or activator and at the same time exposed to fluoride. The enrichment of gene signaling pathways related to autophagy was evaluated by Gene Set Enrichment Analysis; expressions of NQO2 and autophagy‐related protein 5 (ATG5), LC3‐II and p62, and mammalian target of rapamycin (mTOR) were quantified by Western‐blotting or fluorescent staining; and the levels of malondialdehyde (MDA) and superoxide dismutase (SOD) assayed biochemically and reactive oxygen species (ROS) detected by flow cytometry.

**Results:**

In the hippocampal CA3 region of rats exposed to high fluoride, the morphological characteristics of neurons were altered; the numbers of autophagosomes in the cytoplasm and the levels of NQO2 increased; the level of p‐mTOR was decreased, and the levels of ATG5, LC3‐II and p62 were elevated; and genes related to autophagy enriched. In vitro, in addition to similar changes in NQO2, p‐mTOR, ATG5, LC3 II, and p62, exposure of SH‐SY5Y cells to fluoride enhanced MDA and ROS contents and reduced SOD activity. Inhibition of NQO2 with RNAi or an inhibitor attenuated the disturbance of the autophagic flux and enhanced oxidative stress in these cells exposed to high fluoride.

**Conclusion:**

Our findings indicate that NQO2 may be involved in regulating autophagy and oxidative stress and thereby exerts an impact on brain injury caused by chronic fluorosis.

## Graphical abstract

We reported for the first time that NQO2 may be involved in regulating autophagy and oxidative stress and thereby exert an impact on brain injury caused by chronic fluorosis. Suppressing NQO2 expression relieves oxidative stress and restores autophagy flux in fluoride‐treated neuron cells.

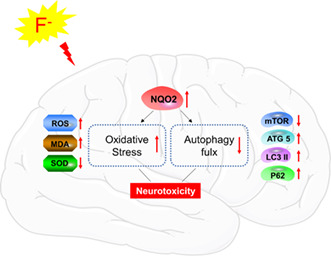

## 1 INTRODUCTION

It has long been known that fluoride can penetrate the blood–brain barrier and damage neuronal cells, thereby causing impairment of learning and memory.^1^, ^2^, ^3^, ^4^ However, the molecular mechanism(s) underlying the effects of endemic (chronic) fluorosis on the central nervous system (CNS) remain to be elucidated.

In this context, on the basis of accumulating evidence is it now becoming widely accepted that an enhanced level of oxidative stress may be involved.^5^, ^6^ The brains of experimental animals exposed to chronic fluorosis contain elevated levels of metabolites indicative of oxidative stress, such as malondialdehyde (MDA), carbonylated proteins, and 8‐hydroxy‐deoxyguanine in DNA, as well as reduced activities of antioxidant enzymes such as superoxide dismutase (SOD), glutathione peroxidase and catalase.^7^, ^8^, ^9^ Moreover, antioxidants effectively alleviate the neuropathological damage and dysfunction resulting from fluorosis.^10^, ^11^ Thus, there is now considerable focus on determining how excess fluoride in brain tissue enhances the level of oxidative stress, as well as the consequences of this enhancement.

Autophagy mediated by lysosomes plays a key role in the maintenance of cellular homeostasis through the strictly regulated degradation and recycling of cellular components.^12^ In eukaryotic cells, the autophagic program is responsible for removing damaged organelles and abnormal proteins^13^ and under varying stressful conditions, the survival of neuronal cells is dependent on this process.^14^ Thus, in addition to its central involvement in the development and normal functioning of brain cells, impairment of autophagy is almost always involved in brain injury as well.^15^, ^16^, ^17^

Interestingly, the reactive oxygen species (ROS) that give rise to oxidative stress can induce autophagy and, at the same time, autophagy can alleviate the damage caused by oxidative stress. When silver nanoparticles (which, due to their antimicrobial activity, are the nanomaterial used most commonly in commercial products) are taken up by murine pro‐B cells (Ba/F3), accumulation of autophagosomes occurs via a process involving activation of mammalian target of rapamycin (mTOR) signaling by ROS.^18^ In addition, when autophagy is induced in CNE‐2Z cells with Earle's balanced salt solution, this process could be inhibited through the suppression of ROS generation by the antioxidant N‐acetyl‐l‐cysteine.^19^ Moreover, autophagy is triggered by the oxidative stress induced by 6‐hydroxydopamine/ascorbic acid, which is a survival mechanism in the lesions of Parkinson's disease (PD).^20^ In a study, euxanthone attenuated the impairment of memory and spatial learning induced by β‐amyloid peptide (Aβ) in an animal model of Alzheimer's disease (AD) and also protected the neuroblastic PC12 cells against Aβ‐induced oxidative stress by promoting autophagy.^21^ Moreover, it was recently demonstrated that both the extent of apoptosis and level of autophagy in retinal astrocytes (RACs) are positively correlated with the level of oxidative stress, which stimulates these cells to release exosomes that activate the proliferation and migration of endothelial cells.^22^ Di‐(2‐ethylhexyl) phthalate (DEHP) impairs testicular and reproductive functions and simultaneously increases the number of autophagosomes and level of microtubule‐associated protein 1A/1B‐light chain 3B (LC3‐II) and p62, markers of autophagy, indicating that autophagosomes accumulate as a result of impaired autophagy.^23^

Recently, abnormal autophagy has been found to be involved in neurotoxicity due to exposure to excessive fluoride, as demonstrated by elevated expression of Beclin1, LC3‐II, and p62 in the hippocampus of rats and in SH‐SY5Y cells caused by such exposure.^24^ In rats, chronic fluorosis impairs learning and memory and simultaneously decreases the number of neurons, suppresses autophagy, and enhances apoptosis in the hippocampus of the brain.^25^ Moreover, the ultrastructure of hippocampal neurons is disrupted in rats exposed to fluoride, perhaps due to more extensive autophagy, as reflected in the enhanced expression of Beclin‐1 in the hippocampal CA1 and DG regions of these animals.^26^

However, the biological mechanisms underlying these effects and processes remain unclear. Using high‐throughput transcriptome sequencing (RNA‐seq) in combination with tandem mass tag, we previously found that the brains of rats subjected to chronic fluorosis are characterized by overexpression of 13 related genes, most of which are primarily involved to oxidative stress and neurodegeneration.^27^ Interestingly, one of the genes up‐regulated by exposure to fluoride encodes the flavoenzyme NRH:quinone oxidoreductase 2 (NQO2), which is involved in regulating both autophagy and oxidative stress.^28^, ^29^

The 231‐amino‐acid NQO2 is localized in the cytosol and uses dihydronicotinamide riboside as an electron donor. The human gene, which exhibits extensive polymorphism, has been mapped precisely to chromosome 6p25.^30^ Expression of NQO2 is elevated in association with neurodegenerative diseases, as exemplified by the abnormally high levels of this enzyme in the hippocampus of patients with AD, which might promote the progression of this disease by increasing the levels of toxic quinones.^31^ Moreover, overexpression of NQO2 in SH‐SY5Y cells exposed to exogenous dopamine elevates the production of ROS, suggesting that higher expression of NQO2 may explain the association between the allelic variant in the D‐promoter of its gene and PD.^32^ The Parkinsonian toxin paraquat (PQ) reduces the basal levels of LC3‐II‐ and LC3‐positive vesicles, as well as their colocalization with lysosomal markers. Interestingly, attenuation of oxidative stress by NMDPEF, a selective inhibitor of NQO2, abrogates the inhibition of autophagy by PQ.^29^

In the current investigation, we examined potential correlations between the levels of NQO2, autophagy, and oxidative stress in the hippocampus of rats subjected to chronic fluorosis, as well as in SH‐SY5Y cells exposed to fluoride. Our findings indicate that NQO2 may be involved in mediating the dysfunction in autophagy and elevated oxidative stress caused by fluorosis.

## 2 MATERIALS AND METHODS

### 2.1 Materials

Sodium fluoride (NaF, S7920), menadione (47775), 4,6‐diamidino‐2‐phenylindole dihydrochloride (DAPI) (28718‐90‐3) and S29434 (MCE, HY‐122614) (Sigma Aldrich); anti‐NQO2 antibody (GR257660‐8) (Santa Cruze); anti‐p‐mTOR (39321), ‐LC3A/B (7074) and ‐rabbit IgG (12741) antibodies (Cell Signaling Technology, Inc.); anti‐autophagy‐related protein 5 (ATG5) (GR3195291‐10), −p62 (GR124843‐69) and ‐GAPDH antibodies (Abcam); mRFP‐GFP‐LC3 adenovirus (Hanbio Biotechnology Co., Ltd.); packaging plasmid system (GV115, Helper 2.0) (Genechem Co., Ltd.); and all other general chemicals (Sigma Aldrich) were purchased from the sources indicated.

Neuroblastoma SH‐SY5Y cells were purchased from the Conservation Genetics CAS Kunming Cell Bank, China. Sprague–Dawley (SD) rats (4 weeks old, initial weight 90–100 g) were purchased from Liaoning Changsheng Biotechnology Co., LTD, China under the license SCXK (Liao)‐2020‐0001.

### 2.2 Exposure of rats to chronic fluorosis and the SH‐SY5Y cell line to fluoride

During acclimation for 1 week, the 96 SD rats (half male and half female) received regular chow and tap water, each containing <0.5 ppm fluoride (F^−^). Subsequently, these rats were divided randomly into four groups of 24 for each fluoride dose, with six rats in each cage and male and female rats housed separately. Thereafter, the control, low‐, medium‐ and high‐dose groups were allowed to drink tap water containing <0.5 (i.e., normal tap water), 5, 50, and 100 ppm F^−^, respectively, for 3 or 6 months. 50 ppm F^−^ corresponds to 3.78 mg/kg,^33^ which is below the maximal tolerable dose in humans (10 mg/kg).^34^ This study protocol was pre‐approved by the Animal Ethics Committee of Guizhou Medical University, China (Approval No. 200868).

SH‐SY5Y cells were exposed for 24 h to six different conditions, that is, control (with no exposure to fluoride), 50 ppm F^−^, 10 μM S29434 (an inhibitor of NQO_2_), 5 μM menadione (an activator of NQO_2_), 50 ppm F^−^ in combination with 10 μM S29434 or 50 ppm F^−^ in combination 5 μM menadione. These conditions were chosen after examining cell survival with the Cell Counting Kit‐8 (Dojindo Laboratories).

### 2.3 Determination of fluoride in serum and brain tissue

At the end of the period of exposure, the rats were anesthetized, blood collected from the heart into a tube that did not contain anticoagulant, and the serum was separated by centrifugation. Then, the animals were sacrificed and brain tissue collected and a portion of it homogenized, with the remainder being stored at −80°C for later use. The levels of F^−^ in serum and brain tissue were determined with a fluoride‐selective electrode.^35^

### 2.4 Examination of the ultrastructure of the hippocampus by transmission electron microscopy

Blocks of tissue (approximately 1 mm^3^ in volume) from the CA3 region of the right hippocampus were fixed in 4% glutaraldehyde, followed by 1% osmium tetroxide in 0.1 mM phosphoric acid buffer (pH 7.4) at room temperature (20°C) for 2 h. These samples were subsequently dehydrated through a series of aqueous alcohol solutions containing a decreasing portion of water and embedded in 812 epoxy resin. Using an ultra‐microtome (Leica UC7), 70 nm‐thick slices were cut, and then these slices were stained with uranium dioxy acetate and lead citrate and examined under a transmission electron microscope (HT 7700; Hitachi).

### 2.5 Assessment of autophagy in the hippocampus using transcriptomic sequencing and Gene Set Enrichment Analysis

Approximately 200 μg of hippocampal tissue was preserved in RNA protection solution and thereafter rapidly cooled in liquid nitrogen and stored at −80°C. The high‐throughput sequencing of these samples performed by Novogene Co., Ltd., China, involved RNA extraction and quality control, library establishment and detection, Illumina sequencing, data quality control, sequence alignment with the reference genome, transcript annotations, and quantification of gene expression. GSEA was used to evaluate the levels of products of genes related to autophagy (RNO04140).

### 2.6 Quantification of NQO2 and proteins related to autophagy in the hippocampus and SH‐SY5Y cells by Western blotting

Proteins extracted from the hippocampal region of rat brains and SH‐SY5Y cells were analyzed by Western blotting. In brief, the total proteins of brain tissue samples and SH‐SY5Y cells were extracted by Total Protein Extraction Kit (Promega), then the total proteins were pooled together by group. Following quantification of the protein extracts with the bicinchoninic acid procedure, the samples were loaded onto 10% polyacrylamide gels, separated by electrophoresis, and subsequently transferred to PVDF membranes. After blocking these membranes for 2 h with Tris‐buffered saline containing Tween 20 (TBST), they were incubated overnight with anti‐NQO2, ‐p‐mTOR, ‐ATG5, ‐LC3A/B, or ‐p62 antibodies and thereafter with anti‐rabbit IgG antibody for 2 h. After ECL staining, specific bands were detected by Biorad Chemiluminescence Analyzer and their densities determined with the Imagine J software, USA. The experiments were performed with three technical replicates.

### 2.7 Determination of autophagic flux in SH‐SY5Y cells using NQO2 RNA interference (viral) as well as NQO2 inhibitor or activator

Based on the sequence of the human NQO2 gene,^36^ three RNAi molecules with a “stem loop” structure and targeting different regions of this gene were designed, synthesized, and then attached to the GV493 vector. This ligation product was transformed into competent *Escherichia coli* for amplification. Following selection with puromycin, resistant colonies were examined by genomic PCR and gene sequencing. Thereafter, to generate lentiviral particles, the recombinant plasmids were co‐transfected with a packaging plasmid system (GV115, Helper 2.0; Genechem Co., Ltd.) into 293 T cells and 48 h later, the resultant viral particles were harvested by centrifugation at 20,000 g for 4 h.

SH‐SY5Y cells were transfected with the three RNAi lentiviruses (2 × 10^9^ particles/ml; MOI = 20) for 8 h, after which the medium was changed to complete medium for 48 h, and screening for stably transfected cells accomplished with puromycin (2 μg/ml). The level of NQO2 mRNA in these SH‐SY5Y cells was evaluated by qRT‐PCR and the level of the corresponding protein quantified by Western blotting. Transection with lentivirus‐mock served as the negative control.

SH‐SY5Y cells were also treated with S29434 or menadione alone or in combination with fluoride exposure.

To allow monitoring of their autophagic flux, SH‐SY5Y cells were cultured in a confocal dish overnight, treated with 50 ppm F^−^ for 48 h and, after changing the culture medium, transfected with the tandem fluorescent mRFP‐GFP‐LC3 adenovirus (1 × 10^9^ PFU/ml). Thereafter, the infected cells were washed with phosphate buffer saline, fixed in 4% paraformaldehyde, mounted with a reagent containing DAPI, and their fluorescence observed under a confocal microscope (FV3000; Olympus).

Red fluorescent protein (RFP) tracks LC3 continuously, while green fluorescent protein (GFP) is an indicator of fusion between autophagosomes and lysosomes. The autophagic flux was evaluated by counting the number of red and green signals.^37^ In the absence of autophagy, the LC3 fusion protein is dispersed through the cytoplasm, without the formation of distinct dots. When autophagosomes form, this protein is localized to the membrane of these structures and points of yellow fluorescence resulting from the overlap of red and green light appear.

When autophagy is proceeding in a normal fashion, autophagosomes and lysosomes fuse to form autolysosomes, in which the acidic environment can quench GFP, so that the number of yellow dots decreases while the number of red dots rises. However, when autophagy is blocked and the autophagosomes cannot fuse with lysosomes, GFP is not quenched and there are more yellow than red dots of fluorescence.

### 2.8 Levels of reactive oxygen species and malondialdehyde and activity of superoxide dismutase in SH‐SY5Y cells exposed to fluoride

Following treatment in one of the manners described above, SH‐SY5Y cells were homogenized and the supernatant was prepared by centrifugation at 4°C. The level of MDA and activity of SOD were determined using commercial kits (Nanjing Jiancheng Bioengineering Int.), while ROS was measured using a DCFDA‐cellular ROS detection kit (Abcam).

### 2.9 Statistical analysis

All data were first assessed for normal (Gaussian) distribution and then analyzed by standard parametric or non‐parametric tests. These values were expressed as means ± SD and the different groups compared by analysis of variance (ANOVA) followed by Dunnett's post‐hoc tests (for normal distribution) or Kruskal–Wallis test (for abnormal distribution). The correlations by Pearson correlation analysis in all cases were used with the GraphPad Prism 8.0 software (GraphPad Software Inc.), and *p*‐value of <0.05 was considered statistically significant difference.

## 3 RESULTS

### 3.1 The level of fluoride in the serum and brain of rats exposed to chronic fluorosis

**FIGURE 1:**
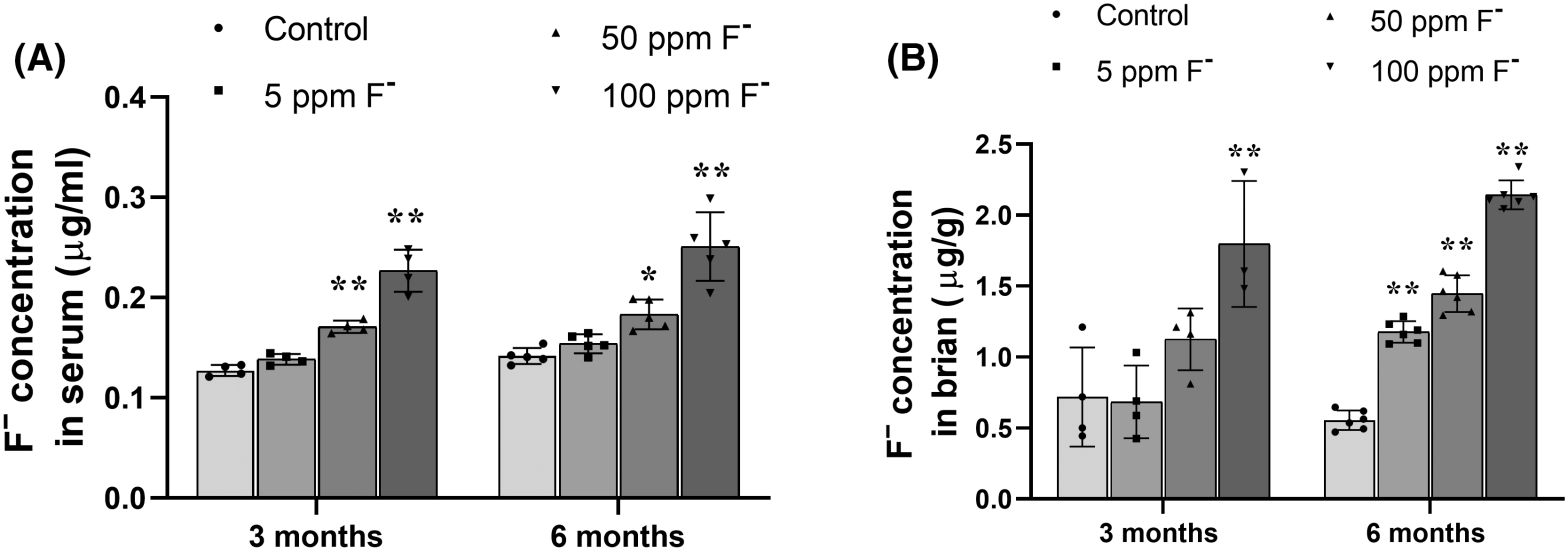
Levels of fluoride in the serum and brain of rats exposed to different levels of fluoride for 3 or 6 months. These levels were determined with a fluoride‐selective electrode. (A) Fluoride content of serum. (B) Fluoride content in brain tissue. The values are expressed as means ± SD. *p < 0.05 and **p < 0.01 in comparison with the untreated (control) group, as determined by analysis of variance (ANOVA) and Dunnett's post‐hoc test.

The content of fluoride in the serum (Figure 1A) and brain (Figure 1B) of rats exposed to different levels of fluoride were elevated in a dose‐ and time‐dependent manner.

### 3.2 Morphological alterations in the CA3 region of the hippocampus of rats exposed to chronic fluorosis

**FIGURE 2:**
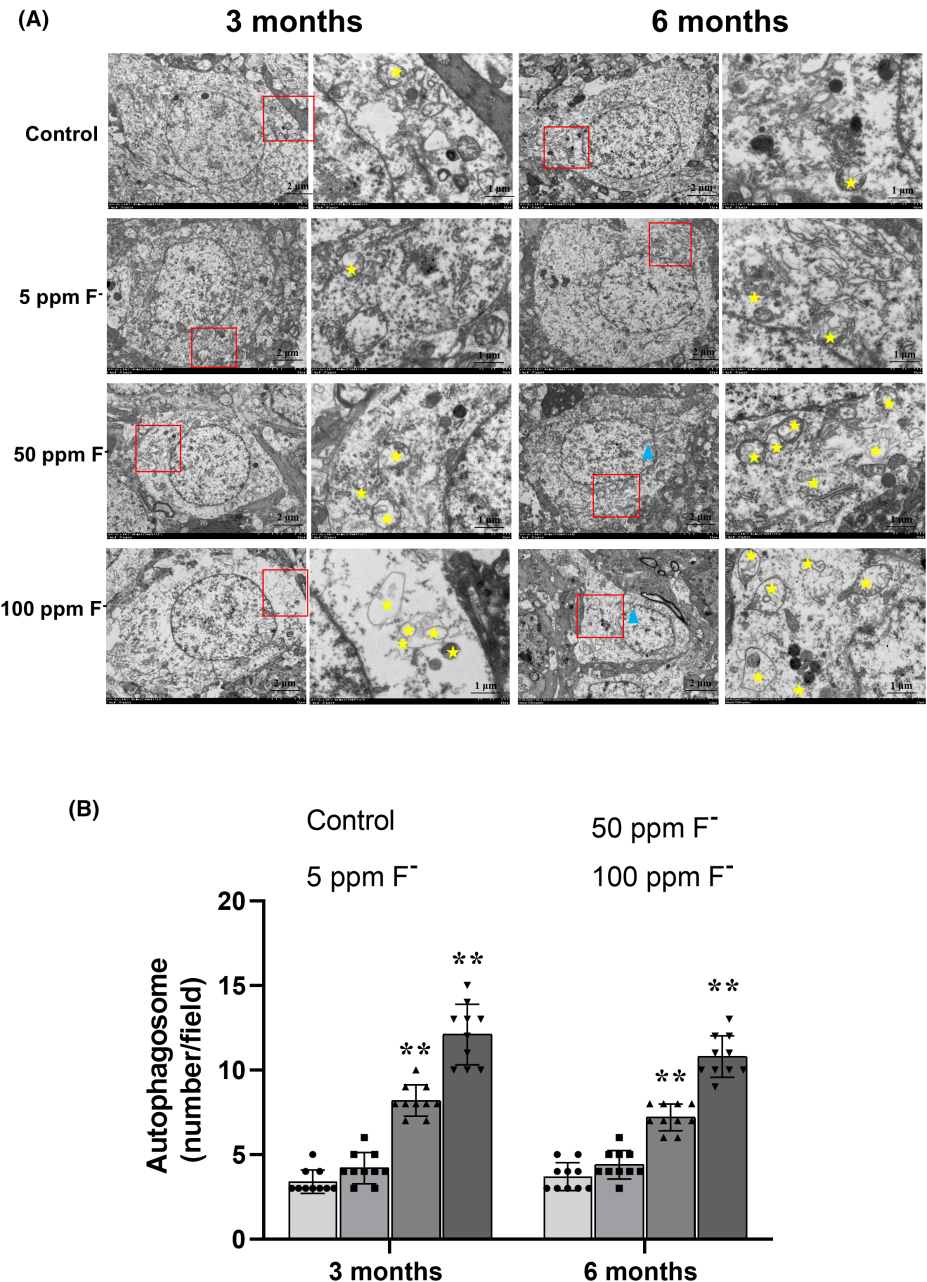
Morphological alterations in the CA3 region of the hippocampus of rats exposed to chronic fluorosis, as observed by transmission electron microscopy (TEM). (A) Ultrastructure of neurons in the hippocampus of rats exposed to fluoride for 3 or 6 months (at magnifications of 2500× and 8000×, respectively). The yellow stars indicate autophagosomes and the blue triangle shrunken nuclei. (B) Quantification of the number of autophagosomes in neurons from the different groups of animals. Under the electron microscope, 10 fields were selected for observation and the values are expressed as means ± SD. **p < 0.01 in comparison with the untreated (control) group, as determined by a two‐way ANOVA and Dunnett's post‐hoc test.

As revealed by transmission electron microscopy (Figure 2), in the neurons of untreated (control) rats, the morphological characteristics of the nucleus were normal and the autophagosomes small. Upon exposure of these animals to the two highest doses of fluoride, the nuclear membrane became to be wrinkled and the chromatin aggregated, while, at the same time, the autophagosomes increased in size. In addition, there were clearly more autophagosomes in the hippocampal neurons of rats exposed to a high level of fluoride.

### 3.3 Changes in the level of proteins and gene signaling activation related to autophagy in the hippocampus of rats exposed to chronic fluorosis

**FIGURE 3:**
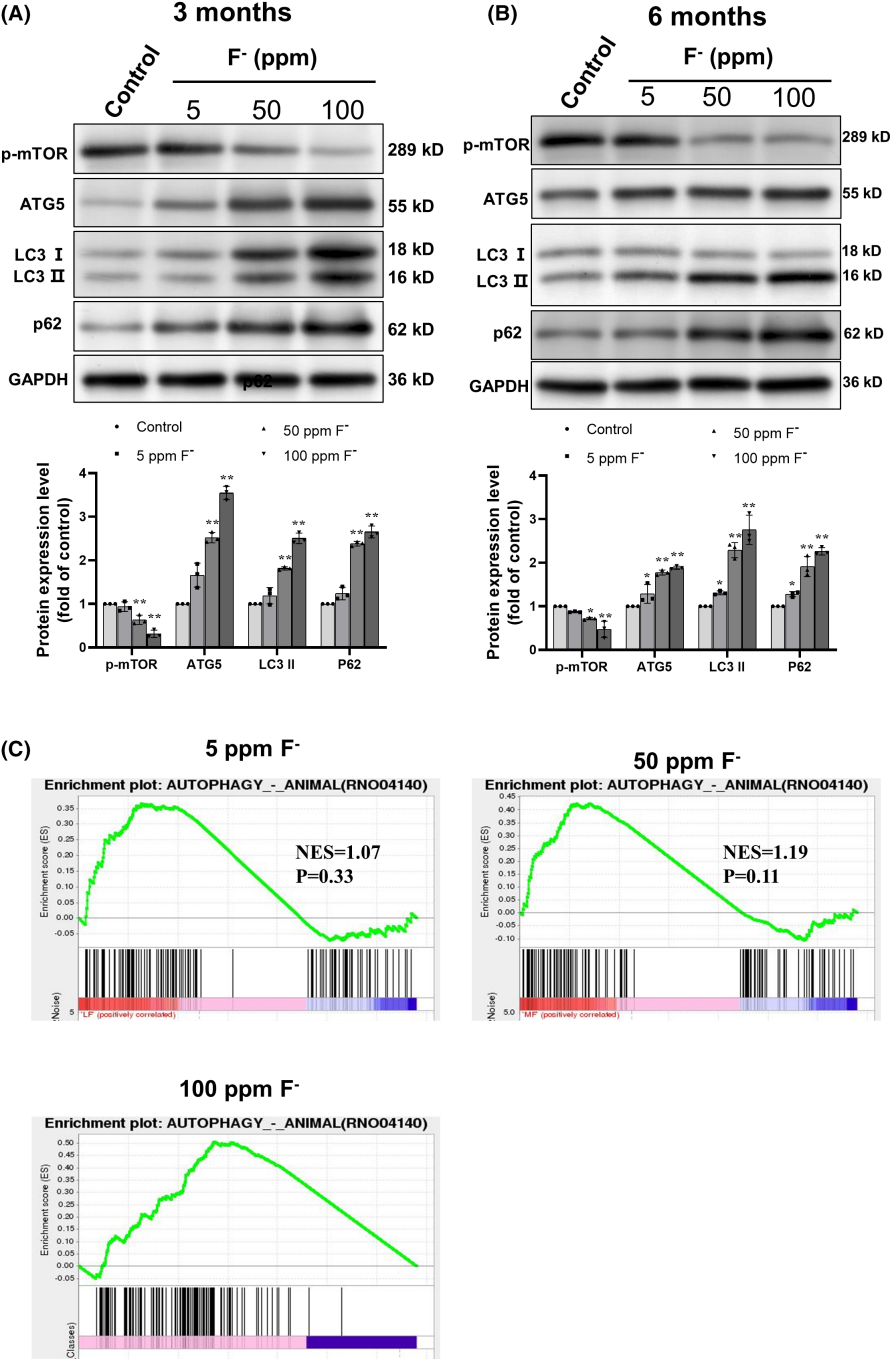
Alterations in the levels of proteins and gene signaling activation related to autophagy in the hippocampus of rats exposed to chronic fluorosis. (A) Western blots of the proteins targeted after 3 months of exposure, along with the housekeeping protein glyceraldehyde phosphate dehydrogenase (GAPDH). (B) Western blots of the proteins targeted after 6 months of exposure. The values are expressed as means ± SD. *p < 0.05 and **p < 0.01 in comparison with the untreated (control) group, as determined by a two‐way ANOVA and Kruskal–Wallis test. (C) Enrichment plots for sets of genes related to autophagy (RNO04140) in the hippocampus of rats exposed to chronic fluorosis, created by Gene Set Enrichment Analysis (GSEA). NES, normalized enrichment score.

To confirm the alterations in autophagy associated with chronic fluorosis, expressions of p‐mTOR, ATG5, LC3II, and p62 in the hippocampus of rats were examined (Figure 3A,B). Exposure to a high level of fluoride attenuated the level p‐mTOR, while increasing those of ATG5 and LC3 II, all of which are related to the elongation of autophagic vesicles. Moreover, with either 50 or 100 ppm F^−^ the level of LC3‐II was higher after 6 than 3 months of exposure. Furthermore, the level of p62 rose in a dose‐dependent manner.

Using the GSEA database for comparison, we found an enriched expression of gene‐encoding components of the autophagy signaling pathway (RNO04140) in the hippocampus of the rat following exposure to a high level of fluoride for 3 months (Figure 3C). The reduction in the nominal *p*‐value with elevated fluoride exposure indicates that this enrichment was dose‐dependent.

### 3.4 Correlation between the level of NQO2 and those of other proteins related to autophagy in the hippocampus of rats exposed to chronic fluorosis

**FIGURE 4:**
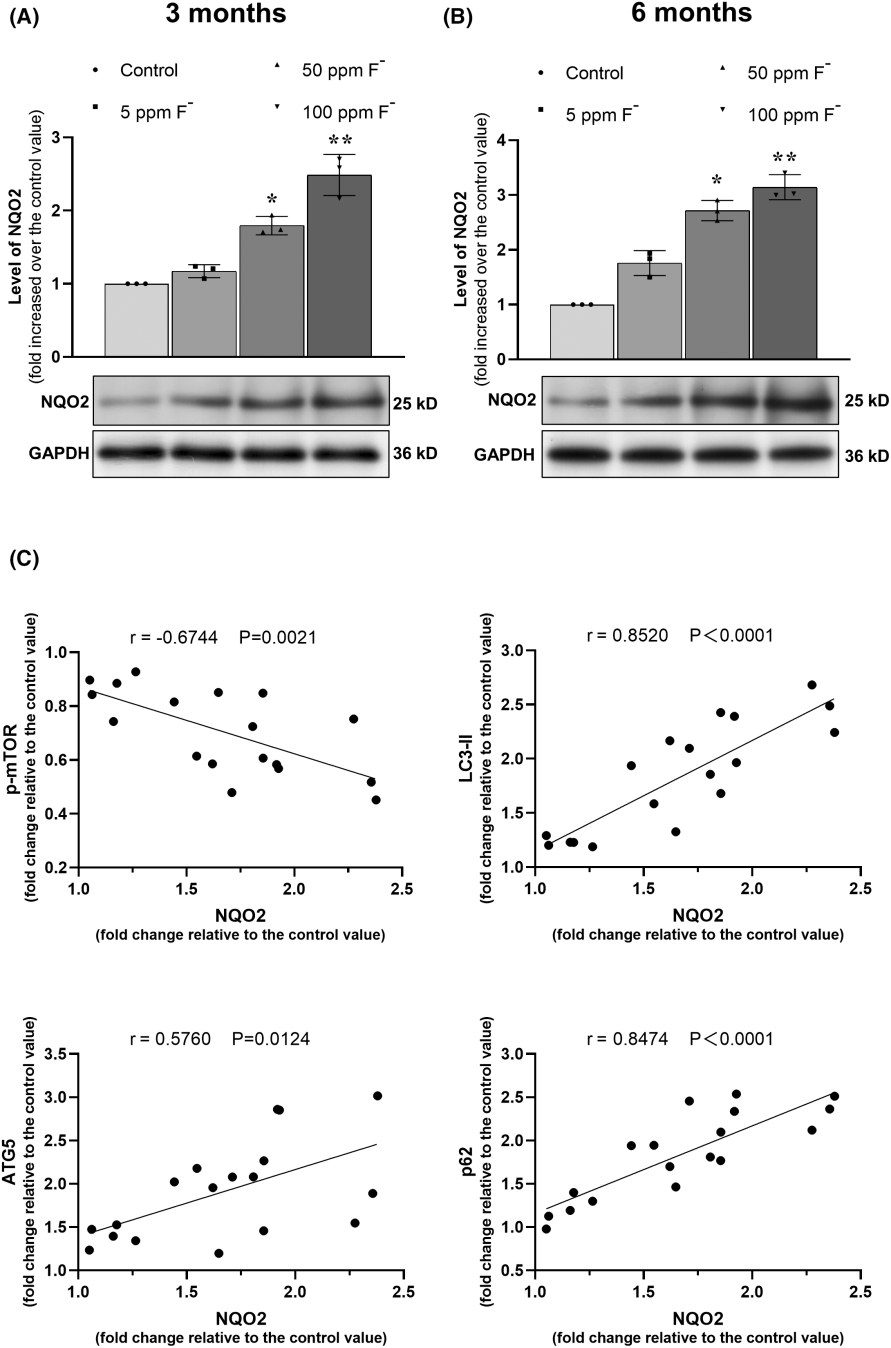
Level of NQO2 in the hippocampus of rats exposed to chronic fluorosis for 3 (A) or 6 months (B), as detected by Western blotting. The values are expressed as means ± SD. *p < 0.05 and **p < 0.01 in comparison with the related groups marked with transverse line as determined by a two‐way ANOVA and Kruskal–Wallis test. Beneath these graphs the representative Western blots for NQO2 and the housekeeping protein glyceraldehyde phosphate dehydrogenase (GAPDH) are shown. Correlations between the level of NQO2 and those of other proteinsrelated to autophagy in the hippocampus of rats exposed to chronic fluorosis (C), as determined by Pearson correlation analysis.

In agreement with our previous report,^27^ we observed here that chronic fluorosis in rats was associated with dose‐ and time‐dependent increases in the level of the NQO2 protein in the brains of these animals (Figure 4A,B). Subsequently, as shown in Figure 4C, Pearson correlation analysis revealed that in rats exposed to chronic fluorosis, the level of NQO2 was negatively correlated with the level of p‐mTOR and positively with the levels of ATG5, LC3 II, and p62.

### 3.5 The influence of NQO2 on autophagy in SH‐SY5Y cells

**FIGURE 5:**
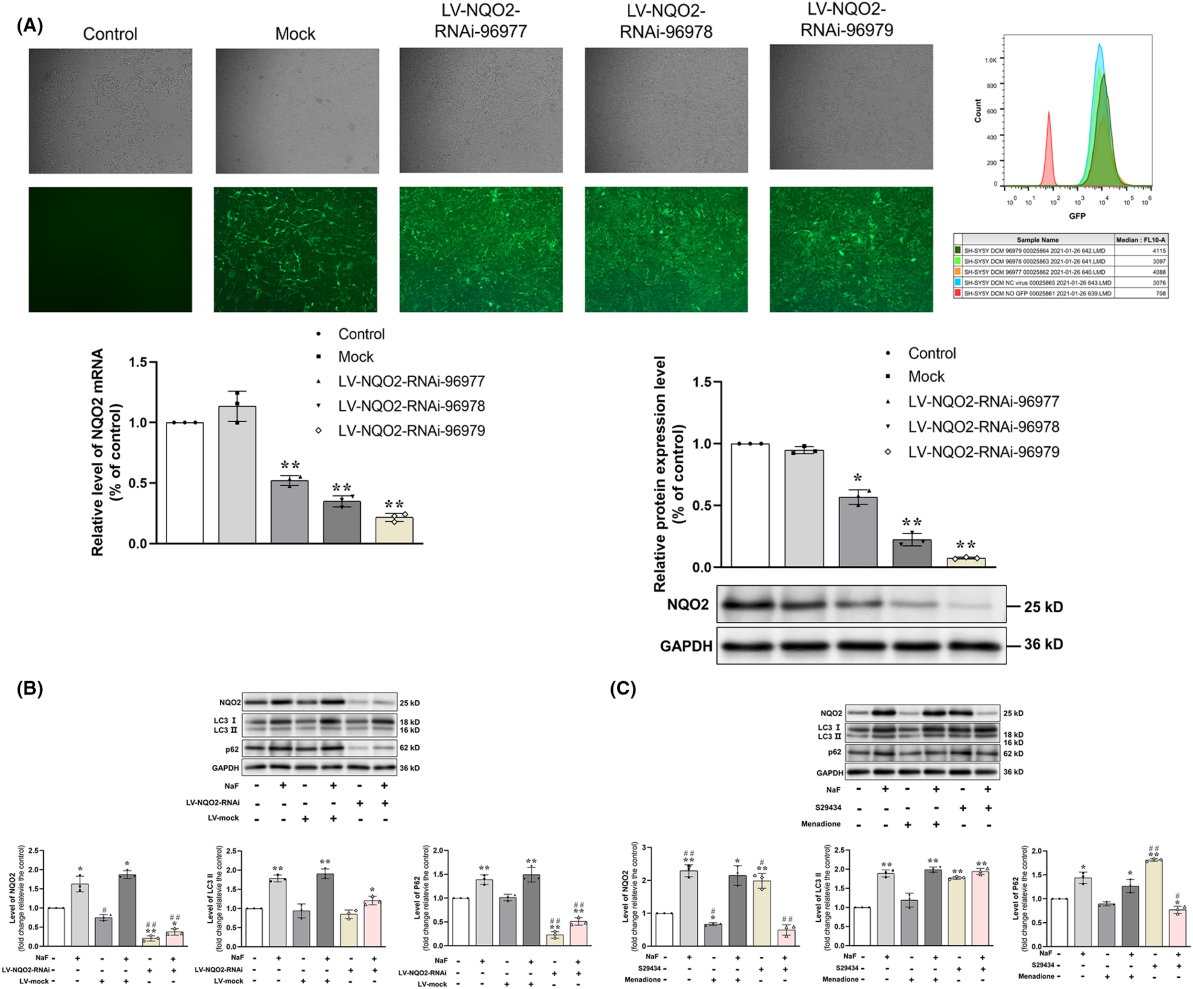
Alterations in the levels of proteins related to autophagy in SH‐SY5Y cells treated with RNAi, S29434, menadione, and/or fluoride. (A) The effects of RNAi lentivirus on the expression of NQO2 in SH‐SY5Y cells. Fluorescence microscopy revealed the expression of green fluorescent protein encoded by the virus 72 h after transfection (100×). The transfection efficiencies were evaluated by Flow Cytometric. Level of NQO2 mRNA and protein in the cells as determined by RT‐PCR and Western blotting analysis. The values are expressed as means ± SD. (B) Levels of proteins related to autophagy in SH‐SY5Y cells transfected with LV‐NQO2 RNAi and then exposed to fluoride. LV‐Mock means transfection with an empty lentivirus and served as the negative control. (C) Alterations in the levels of proteins related to autophagy in SH‐SY5Y cells treated with S29434, menadione and/or fluoride. The values are expressed as means ± SD. *p < 0.05 and **p < 0.01 in comparison with the correspondent control, and ^##^ *p* < 0.01 compared with the correspondent group exposed to fluoride, as determined by a two‐way ANOVA and Kruskal–Wallis test.

In an attempt to unravel the mechanism by which NQO2 is related to the changes in autophagy and oxidative stress induced by fluorosis, we applied RNAi and small molecular inhibitor and activator to alter gene expression and protein level of NQO2 on SH‐SY5Y cells (Figure 5A). Successful transfection was confirmed by both fluorescence microscopy and flow cytometry. Moreover, using qRT‐PCR and Western blotting, we verified that the levels of both NQO2 mRNA and protein were lowered significantly by RNAi, to the greatest extent by NQO2‐RNAi‐96979.

Subsequently, when SH‐SY5Y cells were transfected with NQO2‐RNAi‐96979 for 48 h, the levels of NQO2 and p62 proteins were significantly lower than those in untransfected cells (Figure 5B). Significantly, the transfection with NQO2‐RNAi‐96979 in SH‐SY5Y cells attenuated the increased level of NQO2 and p62 proteins induced by exposure of 50 ppm F^−^. However, this interference with NQO2 expression did not obviously alter the level of LC3 resulted from exposure of fluoride.

To further confirm the involvement of NQO2 in regulating autophagy in SH‐SY5Y cells, we applied an inhibitor and activator of NQO2, namely S29434 and menadione, respectively, with and without exposure to fluoride (Figure 5C). S29434 lowered the levels of NQO2 and p62 but did not alter the level of LC3II, a pattern similar to the effects of transfection with NQO2 RNAi. On the other hand, menadione increased the levels of all three of these proteins. Thus, in these cells, S29434 attenuated, while menadione augmented the effects of exposure to fluoride on the levels of proteins related to autophagy.

### 3.6 The influence of inhibition or activation of NQO2 on the enhancement of autophagy and the level of oxidative stress in SH‐SY5Y cells caused by exposure to fluoride

**FIGURE 6:**
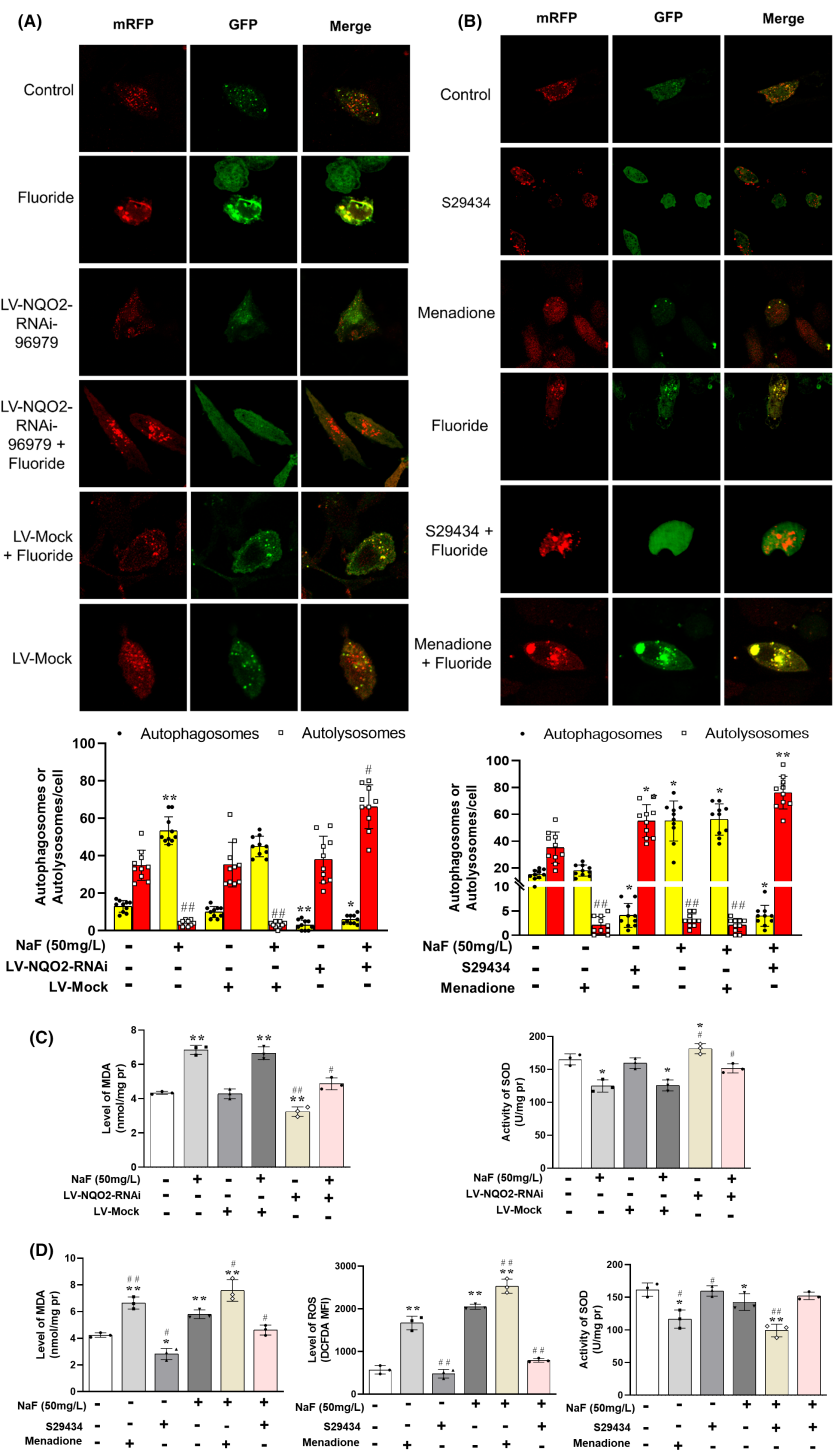
Alterations in the autophagic flux and oxidative stress in SH‐SY5Y cells transfected with LV‐NQO2 RNAi or treated with S29434 or menadione in combination with exposure to fluoride. These changes revealed by transfection with the mRFP‐GFP‐LC3 adenovirus were observed under the confocal microscope. LV‐Mock means transfection with the empty lentivirus and served as a negative control. (A) Representative confocal fluorescent images following treatment of the cells with NQO2 RNAi and fluoride (600×). Quantification of autophagosomes (yellow) and autolysosomes in transfected cells. (B) Representative confocal fluorescent images following treatment of the cells with S29434 or menadione in combination with fluoride (600×). Quantification of autophagosomes (yellow) and autolysosomes (red) in transfected cells or the cells treated with S29434 or menadione in combination with fluoride. (C) Alterations in the level of oxidative stress in SH‐SY5Y cells following transfection with NQO2 RNAi or D. treatment with S29434 or menadione in combination with exposure to fluoride. The values are expressed as means ± SD. *p < 0.05 and **p < 0.01 in comparison with the corresponding controls, and ^#^ *p* < 0.05 and ^##^ *p* < 0.01 in comparison with the correspondent group exposed to fluoride, as determined by a two‐way ANOVA and Kruskal–Wallis test.

In Figure 6A,B, untransfected cells not exposed to fluoride contained more red than yellow fluorescent dots, indicating that autophagy was proceeding in a normal manner. Following exposure of these cells to fluoride, there was more formation of autophagosomes, as reflected in a larger number of red dots. However, very few autolysosomes were formed, so that the green fluorescence was not extensively quenched and yellow fluorescent dots dominated. This pattern is indicative of arrested autophagy.

Interference with NQO2 expression in SH‐SY5Y cells by transfection with RNAi reduced the formation of autophagosomes, but a larger number of autolysosomes were formed and quenched green dots, resulting in more red fluorescent dots in the overlap pattern (Figure 6A). With these cells, the arrest in autophagy caused by fluoride was attenuated and red dots dominated in a manner similar to the pattern observed with the negative control (LV‐mock) and untransfected cells exposed to fluoride.

In addition, treatment of unexposed SH‐SY5Y cells with S29434 altered the pattern of fluorescence in a manner similar to the effects of NQO2 interference (Figure 6B). At the same, exposure of unexposed cells to both this inhibitor and fluoride attenuated the effects of the latter on autophagy, resulting in more abundant autophagosomes and autolysosomes, as well as an overlap pattern dominated by red fluorescent dots. On the other hand, exposure of these same cells to both menadione and fluoride blocked the formation of autolysosomes and yellow dots became dominant in the overlap map.

Exposure of SH‐SY5Y cells to fluoride increased their levels of MDA and ROS and reduced their SOD activity, while reduction of NQO2 with RNAi attenuated these indicators of enhanced oxidative stress (Figure 6C). Chemical inhibition of NQO2 had similar effects, whereas activation of this enzyme augmented the level of oxidative stress caused by fluoride (Figure 6D).

## 4 DISCUSSION

Here, rats drinking water containing high levels of fluoride for 3 or 6 months exhibited significantly elevated fluoride contents in their serum and brains. This clearly indicates that fluoride can be taken up from the gastrointestinal tract into the blood and subsequently cross the blood–brain barrier.^27^ In addition, cultured SH‐SY5Y cells exposed to fluoride demonstrated elevated levels of oxidative stress.

During the initial stage of autophagy, several members of the ATG family of proteins play central roles in the formation of large complexes located on the outside of the membrane of the phagophore. These complexes activate the enzyme ATG3, which catalyzes covalent conjugation of LC3 to the amino group of phosphatidylethanolamine (PE), a key step in autophagosome formation.^38^ During the process of phagophore extension and autophagosome formation, LC3 I binds to PE via a ubiquitin‐like reaction to produce lipidized LC3 II, an important marker on the membrane of the autophagosome. Interestingly, the level of LC3 II in the substantia nigra of patients with PD is elevated significantly.^39^ Meanwhile, another key molecule, P62, binds to ubiquitinated proteins and forms a complex with LC3 II.^12^ Importantly, during the normal process of autophagy, P62 is degraded.

As mentioned earlier, it is now well established that chronic fluorosis results in pathological damage to the CNS and cognitive dysfunction.^6^, ^40^ Neurons are extremely polarized and require a steady supply of nutrients and energy, while relying heavily on autophagy to remove harmful compounds and prevent the accumulation of metabolic waste products.^41^ The brain damage induced by fluorosis is associated with impaired autophagy, as reflected in the elevated levels of Beclin1, LC3‐II, and p62 in the hippocampus of rats with chronic fluorosis, as well as in SH‐SY5Y cells exposed to fluoride.^24^ Inhibition of mTOR with rapamycin protects SH‐SY5Y cells from defective autophagy and excessive apoptosis, thereby enhancing their survival.^25^

In a previous investigation, we observed dysregulation of the balance between mitochondrial fission and fusion in the hippocampus of rats exposed to chronic fluorosis.^42^ Such abnormal mitochondrial dynamics can trigger abnormal levels of autophagy and apoptosis, leading to neuronal death.^43^ Autophagy in the hippocampus of rats exposed to chronic fluorosis occurs mainly in the CA1 and DG regions of the brain, which may be related to the accumulation of lipofuscin in these same regions.^26^

In the current morphological and biomolecular investigation, we confirm that excessive exposure of rats to fluoride disrupts autophagy. Transmission electron microscopy revealed a wrinkled nuclear membrane and chromatin aggregation in the neurons in the C3 region of the hippocampus of rats exposed to chronic fluorosis. This enhanced volume of autophagosomes in the neuronal cytoplasm was correlated to increased exposure to fluoride.

It has been reported that fluorosis promotes autophagy while, at the same time, disrupting this process, as reflected in the change in the autophagy‐inducing factor mTOR, the elevated levels of intermediate proteins such as ATG5, LC3 II, and Beclin1, and the accumulation of P62.^25^, ^44^, ^45^ ATG5 and LC3‐II appear to be indicators of the initiation of autophagy, whereas the level of p62 reflects the extent of autophagic degradation.^46^ Here, the enrichment in autophagy genes we detected using the GSEA database indicated the presence of elevated levels of ATG5, LC3 II, and p62 and less p‐mTOR in the hippocampus of rats exposed to chronic fluorosis, suggesting stimulation of the initial steps of autophagy, but subsequent blockage that was both time‐ and dose‐dependent.

Previously, we used high‐throughput sequencing to monitor changes in the transcriptome and proteome of the hippocampus of rats with chronic fluorosis and identified 13 genes/proteins that demonstrated consistent changes in expression.^27^ These included NQO2, which is associated with neurodegenerative changes, oxidative stress, and autophagy.^28^ Accordingly, we attempted to determine in the present study whether the level of NQO2 is altered in connection with chronic fluorosis and, if so, whether this change is involved in the regulation of autophagy and pathogenesis of brain injury.

NQO2 may well play a role in regulating autophagy and oxidative stress. Treatment with the PQ prevents inhibition of mTOR and upregulation of BECN1/Beclin 1, changes typically associated with the induction of autophagy.^29^ Reduction of oxidative stress by NMDPEF, a selective inhibitor of NQO2, abrogated this inhibition of autophagic flux by PQ. Moreover, overexpression of NQO2 appears to be related to tumor formation, since such overexpression can lead to disruption of the gene encoding p53,^47^ alter signaling via the nuclear factor‐κB pathway^48^ and induce prostate cancer metastasis,^49^ among other effects. The elevated level of NQO2 in the hippocampus, amygdala, and superior frontal gyrus of patients with AD is related to the production of ROS.^31^

Our present findings confirm that exposure of rats to fluoride elevates the expression of the NQO2 protein in the brain in a dose‐dependent manner. We also found that in SH‐SY5Y cells exposed to fluoride in culture, this increased expression was negatively correlated to the level of mTOR and positively correlated to the levels of the autophagy‐related factors ATG5, LC3 II, and P62. These results indicate that fluoride can promote autophagosome formation in the initial stages of autophagy, while causing accumulation of P62, i.e., disrupting autophagic degradation, during later stages.

For elucidating the mechanism(s) underlying these relationships, we transfected SH‐SY5Y cells with NQO2 RNAi or treated these cells with an inhibitor or activator of this enzyme in combination with fluoride. Interfering with the expression of NQO2 or chemically inhibiting this enzyme attenuated the increase in P62 protein induced by fluorosis, indicating that the deficit in autophagic degradation was also attenuated. In contrast, chemical activation of NQO2 activator enhanced this response to fluorosis. At the same time, the elevated level of LC3 II associated with fluorosis was enhanced by activation of NQO2 but clearly not affected by transfection with RNAi or chemical inhibition. P62, also called sequestosome1, is the selective receptor for misfolded proteins destined to undergo autophagic degradation. Ectopic accumulation of p62 in the brain of APP/PS1 mouse models of AD is associated with impaired autophagic flux.^50^ In aged mice, increased expression of LC3 and p62 by cervical motor neurons is indicative of impaired autophagy, with the accumulation of autophagosomes.^51^ In selenium‐deficient trophoblasts, up‐regulation of LC3II, Beclin1, and p62 indicates a dysfunctional autophagic flux.^52^ Our observation indicates that, significantly, NQO2 may regulate the expression of p62, which influences autolysosome formation and autophagic degradation.

To further confirm the influence of NQO2 on the autophagic flux in cultured SH‐SY5Y cells exposed to fluoride, these cells were transfected with the tandem fluorescent MRFP‐GFP‐LC3 adenovirus to allow visualization of autophagosomes and autolysosomes. Exposure to fluoride stimulated the formation of the former, while eliminating the formation of the latter, thereby blocking the autophagic flux. Interference of NQO2 expression by RNAi reduced this formation of autophagosomes and led to more autolysosomes, thereby weakening these effects of fluoride. On the other hand, the agonist of NQO2 enhanced these effects. In addition, both NQO2 silencing and chemical inhibition reduced the level of P62 and attenuated the effect of fluoride on the autophagic flux.

Oxidative stress is an important mediator of brain injury resulting from chronic fluorosis.^7^, ^53^ Appropriate autophagy can protect against injury during the early stages of ischemic stroke, whereas excessive autophagy is correlated with inflammation and oxidative stress.^54^ Here, we show that exposure of SH‐SY5Y cells to fluoride increases their levels of MDA and ROS while lowering the activity of SOD activity. Lowering the activity of NQO2 with RNAi or by chemical inhibition attenuates this elevation of oxidative stress, whereas activation of NQO2 has the opposite effect. We, therefore, speculate that upon exposure to a high level of fluoride, NQO2 regulates autophagy, thus influencing the level of oxidative stress that can lead to injury of the CNS.

In conclusion, in rats exposed to chronic fluorosis, autophagy in the hippocampus was abnormal, as reflected in the lowered expression of p‐mTOR, increased levels of ATG5 and LC3 II, and accumulation of p62. Similar results were obtained when cultured SH‐SY5Y cells were exposed to fluoride. Moreover, fluoride also increased the level of the NQO2 protein in both the brain of rats and in these nerve cells in culture, as well as enhancing oxidative stress in vitro. Inhibition of NQO2 reduced the impairment in the autophagic flux and attenuated the oxidative stress caused by fluorosis. These results indicate that NQO2 may be a regulator of the autophagic flux, thereby influencing oxidative stress in the pathogenesis of brain injury resulting from fluorosis.

## AUTHOR CONTRIBUTIONS

Conceptualization: Long‐Yan Ran; Methodology: Long‐Yan Ran, Jie Xiang, Xiao‐Xiao Zeng, and Wen‐Wen He; Data curation: Yang‐Ting Dong and Jian Zou; Formal analysis and investigation: Long‐Yan Ran and Jie Xiang; Resources: Zhi‐Zhong Guan, Wen‐Feng Yu, Xiao‐Lan Qi, Yan Xiao, and Kun Cao; Writing – original draft preparation: Long‐Yan Ran; Writing – review and editing: Zhi‐Zhong Guan; Funding acquisition: Zhi‐Zhong Guan; Supervision: Zhi‐Zhong Guan.

## CONFLICT OF INTEREST

The authors confirm that there are no conflicts of interest.

## Data Availability

The data that support the findings of this study are available on request from the corresponding author (Zhi‐Zhong Guan) upon reasonable request.

## References

[1] Sharma P , Verma PK , Sood S , Singh R , Gupta A , Rastogi A . Distribution of fluoride in plasma, brain, and bones and associated oxidative damage after induced chronic fluorosis in Wistar rats. Biol Trace Elem Res. 2022;200:1710‐1721. 34128210 10.1007/s12011-021-02782-3

[2] Zeng XX , Deng J , Xiang J , et al. Protections against toxicity in the brains of rat with chronic fluorosis and primary neurons exposed to fluoride by resveratrol involves nicotinic acetylcholine receptors. J Trace Elem Med Biol. 2020;60:126475. 32142957 10.1016/j.jtemb.2020.126475

[3] Guan ZZ , Wang YN , Xiao KQ , et al. Influence of chronic fluorosis on membrane lipids in rat brain. Neurotoxicol Teratol. 1998;20:537‐542. 9761592 10.1016/s0892-0362(97)00136-0

[4] Mullenix PJ , Denbesten PK , Schunior A , Kernan WJ . Neurotoxicity of sodium fluoride in rats. Neurotoxicol Teratol. 1995;17:169‐177. 7760776 10.1016/0892-0362(94)00070-t

[5] Ferreira MKM , Aragão WAB , Bittencourt LO , et al. Fluoride exposure during pregnancy and lactation triggers oxidative stress and molecular changes in hippocampus of offspring rats. Ecotoxicol Environ Saf. 2021;208:111437. 33096359 10.1016/j.ecoenv.2020.111437

[6] Gui CZ , Ran LY , Li JP , Guan ZZ . Changes of learning and memory ability and brain nicotinic receptors of rat offspring with coal burning fluorosis. Neurotoxicol Teratol. 2010;32:536‐541. 20381606 10.1016/j.ntt.2010.03.010

[7] Lopes GO , Ferreira MKM , Davis L , et al. Effects of fluoride long‐term exposure over the cerebellum: global proteomic profile, oxidative biochemistry, cell density, and motor behavior evaluation. Int J Mol Sci. 2020;21:1‐20. 10.3390/ijms21197297 PMC7582550 33023249

[8] Gao Q , Liu YJ , Guan ZZ . Oxidative stress might be a mechanism connected with the decreased α7 nicotinic receptor influenced by high‐concentration of fluoride in SH‐SY5Y neuroblastoma cells. Toxicol Vitr. 2008;22:837‐843. 10.1016/j.tiv.2007.12.017 18282683

[9] Zhang M , Wang A , He W , et al. Effects of fluoride on the expression of NCAM, oxidative stress, and apoptosis in primary cultured hippocampal neurons. Toxicology. 2007;236:208‐216. 17537562 10.1016/j.tox.2007.04.007

[10] Sarkar C , Pal S , Das N , Dinda B . Ameliorative effects of oleanolic acid on fluoride induced metabolic and oxidative dysfunctions in rat brain: experimental and biochemical studies. Food Chem Toxicol. 2014;66:224‐236. 24468673 10.1016/j.fct.2014.01.020

[11] Reddy KP , Sailaja G , Krishnaiah C . Protective effects of selenium on fluoride induced alterations in certain enzymes in brain of mice. J Environ Biol. 2009;30:859‐864. 20143719

[12] Parzych KR , Klionsky DJ . An overview of autophagy: morphology, mechanism, and regulation. Antioxidants Redox Signal. 2014;20:460‐473. 10.1089/ars.2013.5371 PMC3894687 23725295

[13] Sun‐Wang JL , Ivanova S , Zorzano A . The dialogue between the ubiquitin‐proteasome system and autophagy: implications in ageing. Ageing Res Rev. 2020;64:101203. 33130248 10.1016/j.arr.2020.101203

[14] Choi I , Zhang Y , Seegobin SP , et al. Microglia clear neuron‐released α‐synuclein via selective autophagy and prevent neurodegeneration. Nat Commun. 2020;11:1386. 32170061 10.1038/s41467-020-15119-w PMC7069981

[15] Elia LP , Mason AR , Alijagic A , Finkbeiner S . Genetic regulation of neuronal progranulin reveals a critical role for the autophagy‐lysosome pathway. J Neurosci. 2019;39:3332‐3344. 30696728 10.1523/JNEUROSCI.3498-17.2019 PMC6788815

[16] Glatigny M , Moriceau S , Rivagorda M , et al. Autophagy is required for memory formation and reverses age‐related memory decline. Curr Biol. 2019;29:435‐448.e8. 30661803 10.1016/j.cub.2018.12.021

[17] Yin Y , Sun G , Li E , Kiselyov K , Sun D . ER stress and impaired autophagy flux in neuronal degeneration and brain injury. Ageing Res Rev. 2017;34:3‐14. 27594375 10.1016/j.arr.2016.08.008 PMC5250579

[18] Zhu L , Guo D , Sun L , et al. Activation of autophagy by elevated reactive oxygen species rather than released silver ions promotes cytotoxicity of polyvinylpyrrolidone‐coated silver nanoparticles in hematopoietic cells. Nanoscale. 2017;9:5489‐5498. 28401217 10.1039/c6nr08188f

[19] Zheng Y , Chen Z , Gu Z , et al. Starvation‐induced autophagy is up‐regulated via ROS‐mediated ClC‐3 chloride channel activation in the nasopharyngeal carcinoma cell line CNE‐2Z. Biochem J. 2019;476:1323‐1333. 30992317 10.1042/BCJ20180979

[20] Zhuang XX , Wang SF , Tan Y , et al. Pharmacological enhancement of TFEB‐mediated autophagy alleviated neuronal death in oxidative stress‐induced Parkinson's disease models. Cell Death Dis. 2020;11:128. 32071296 10.1038/s41419-020-2322-6 PMC7028954

[21] Yuan H , Jiang C , Zhao J , et al. Euxanthone attenuates Aβ1‐42‐induced oxidative stress and apoptosis by triggering autophagy. J Mol Neurosci. 2018;66:51223‐51523. 10.1007/s12031-018-1175-2 30345461

[22] Zhu L , Zang J , Liu B , et al. Oxidative stress‐induced RAC autophagy can improve the HUVEC functions by releasing exosomes. J Cell Physiol. 2020;235:7392‐7409. 32096219 10.1002/jcp.29641 PMC7496456

[23] Yi WEI , Xiang‐Liang T , Yu Z , et al. DEHP exposure destroys blood‐testis barrier (BTB) integrity of immature testes through excessive ROS‐mediated autophagy. Genes Dis. 2018;5:263‐274. 30320191 10.1016/j.gendis.2018.06.004 PMC6176266

[24] Niu Q , Chen J , Xia T , et al. Excessive ER stress and the resulting autophagic flux dysfunction contribute to fluoride‐induced neurotoxicity. Environ Pollut. 2018;233:889‐899. 29100748 10.1016/j.envpol.2017.09.015

[25] Zhou G , Tang S , Yang L , et al. Effects of long‐term fluoride exposure on cognitive ability and the underlying mechanisms: role of autophagy and its association with apoptosis. Toxicol Appl Pharmacol. 2019;378:114608. 31173788 10.1016/j.taap.2019.114608

[26] Zhang C , Huo S , Fan Y , Gao Y , Yang Y , Sun D . Autophagy may be involved in fluoride‐induced learning impairment in rats. Biol Trace Elem Res. 2020;193:502‐507. 31111310 10.1007/s12011-019-01735-1

[27] Ran LY , Xiang J , Zeng XX , Tang JL , Dong YT , Zhang F . Integrated transcriptomic and proteomic analysis indicated that neurotoxicity of rats with chronic fluorosis may be in mechanism involved in the changed cholinergic pathway and oxidative stress. J Trace Elem Med Biol. 2021;64:126688. 33260044 10.1016/j.jtemb.2020.126688

[28] Janda E , Martino C , Riillo C , et al. Apigenin and luteolin regulate autophagy by targeting nrh‐quinone oxidoreductase 2 in liver cells. Antioxidants. 2021;10:776. 34068281 10.3390/antiox10050776 PMC8153271

[29] Janda E , Lascala A , Carresi C , et al. Parkinsonian toxin‐induced oxidative stress inhibits basal autophagy in astrocytes via NQO2/quinone oxidoreductase 2: implications for neuroprotection. Autophagy. 2015;11:1063‐1080. 26046590 10.1080/15548627.2015.1058683 PMC4590600

[30] Long DJ , Jaiswal AK . NRH:quinone oxidoreductase2 (NQO2). Chem Biol Interact. 2000;129:99‐112. 11154737 10.1016/s0009-2797(00)00200-3

[31] Hashimoto T , Nakai M . Increased hippocampal quinone reductase 2 in Alzheimer's disease. Neurosci Lett. 2011;502:10‐12. 21803122 10.1016/j.neulet.2011.07.008

[32] Wang W , Le WD , Pan T , Stringer JL , Jaiswal AK . Association of NRH:quinone oxidoreductase 2 gene promoter polymorphism with higher gene expression and increased susceptibility to Parkinson's disease. J Gerontol A Biol Sci Med Sci. 2008;63:127‐134. 18314446 10.1093/gerona/63.2.127

[33] Nair A , Jacob S . A simple practice guide for dose conversion between animals and human. J Basic Clin Pharm. 2016;7:27‐31. 27057123 10.4103/0976-0105.177703 PMC4804402

[34] Institute of Medicine (US) Standing Committee on the Scientific Evaluation of Dietary Reference Intakes . Dietary Reference Intakes for Calcium, Phosphorus, Magnesium, Vitamin D, and Fluoride. National Academies Press (US); 1997:306. 23115811

[35] Reshetnyak VY , Nesterova OV , Admakin OI , et al. Evaluation of free and total fluoride concentration in mouthwashes via measurement with ion‐selective electrode. BMC Oral Health. 2019;19:251. 31747894 10.1186/s12903-019-0908-0 PMC6868805

[36] Mocellin S , Provenzano M . RNA interference: learning gene knock‐down from cell physiology. J Transl Med. 2004;2:39. 15555080 10.1186/1479-5876-2-39 PMC534783

[37] Zhou C , Zhong W , Zhou J , et al. Monitoring autophagic flux by an improved tandem fluorescent‐tagged LC3 (mTagRFP‐mWasabi‐LC3) reveals that high‐dose rapamycin impairs autophagic flux in cancer cells. Autophagy. 2012;8:1215‐1226. 22647982 10.4161/auto.20284

[38] Ye Y , Tyndall ER , Bui V , et al. An N‐terminal conserved region in human Atg3 couples membrane curvature sensitivity to conjugase activity during autophagy. Nat Commun. 2021;12:374. 33446636 10.1038/s41467-020-20607-0 PMC7809043

[39] Alvarez‐Erviti L , Rodriguez‐Oroz MC , Cooper JM , et al. Chaperone‐mediated autophagy markers in Parkinson disease brains. Arch Neurol. 2010;67:1464‐1472. 20697033 10.1001/archneurol.2010.198

[40] Dong YT , Wang Y , Wei N , Zhang QF , Guan ZZ . Deficit in learning and memory of rats with chronic fluorosis correlates with the decreased expressions of M1 and M3 muscarinic acetylcholine receptors. Arch Toxicol. 2015;89:1981‐1991. 25417050 10.1007/s00204-014-1408-2

[41] Stavoe AKH , Holzbaur ELF . Autophagy in neurons. Annu Rev Cell Dev Biol. 2019;35:477‐500. 31340124 10.1146/annurev-cellbio-100818-125242 PMC6996145

[42] Lou DD , Guan ZZ , Liu YJ , et al. The influence of chronic fluorosis on mitochondrial dynamics morphology and distribution in cortical neurons of the rat brain. Arch Toxicol. 2013;87:449‐457. 23007560 10.1007/s00204-012-0942-z

[43] Zhao Q , Niu Q , Chen J , et al. Roles of mitochondrial fission inhibition in developmental fluoride neurotoxicity: mechanisms of action in vitro and associations with cognition in rats and children. Arch Toxicol. 2019;93:709‐726. 30659323 10.1007/s00204-019-02390-0

[44] Wang Y , Li A , Mehmood K , et al. Long‐term exposure to the fluoride blocks the development of chondrocytes in the ducks: the molecular mechanism of fluoride regulating autophagy and apoptosis. Ecotoxicol Environ Saf. 2021;217:112225. 33864983 10.1016/j.ecoenv.2021.112225

[45] Feng Z , Liang C , Manthari RK , Wang C , Zhang J . Effects of fluoride on autophagy in mouse Sertoli cells. Biol Trace Elem Res. 2019;187:499‐505. 29915883 10.1007/s12011-018-1405-z

[46] Zhang S , Niu Q , Gao H , et al. Excessive apoptosis and defective autophagy contribute to developmental testicular toxicity induced by fluoride. Environ Pollut. 2016;212:97‐104. 26840522 10.1016/j.envpol.2016.01.059

[47] Da YK , Di GH , Yuan WT , et al. Functional polymorphisms, altered gene expression and genetic association link NRH:quinone oxidoreductase 2 to breast cancer with wild‐type p53. Hum Mol Genet. 2009;18:2502‐2517. 19351655 10.1093/hmg/ddp171

[48] Chariot A . 20 years of NF‐κB. Biochem Pharmacol. 2006;72:1051‐1053. 16999938 10.1016/j.bcp.2006.08.023

[49] Dorai T , Shah A , Summers F , et al. NRH:quinone oxidoreductase 2 (NQO2) and glutaminase (GLS) both play a role in large extracellular vesicles (LEV) formation in preclinical LNCaP‐C4‐2B prostate cancer model of progressive metastasis. Prostate. 2018;78:1181‐1195. 30009389 10.1002/pros.23693

[50] Xu C , Wu J , Wu Y , et al. TNF‐α‐dependent neuronal necroptosis regulated in Alzheimer's disease by coordination of RIPK1‐p62 complex with autophagic UVRAG. Theranostics. 2021;11:9452‐9469. 34646380 10.7150/thno.62376 PMC8490500

[51] Pareja‐Cajiao M , Gransee HM , Stowe JM , Rana S , Sieck GC , Mantilla CB . Age‐related impairment of autophagy in cervical motor neurons. Exp Gerontol. 2021;144:111193. 33290859 10.1016/j.exger.2020.111193 PMC7968728

[52] Li M , Cheng W , Zhang L . Maternal selenium deficiency suppresses proliferation, induces autophagy dysfunction, apoptosis in the placenta of mice. Metallomics. 2021;13:mfab058. 34669944 10.1093/mtomcs/mfab058

[53] Zhang KL , Lou DD , Guan ZZ . Activation of the AGE/RAGE system in the brains of rats and in SH‐SY5Y cells exposed to high level of fluoride might connect to oxidative stress. Neurotoxicol Teratol. 2015;48:49‐55. 25666879 10.1016/j.ntt.2015.01.007

[54] Deng M , Zhong X , Gao Z , et al. Dynamic changes in Beclin‐1, LC3B and p62 at various time points in mice with temporary middle cerebral artery occlusion and reperfusion (tMCAO). Brain Res Bull. 2021;173:124‐131. 33974897 10.1016/j.brainresbull.2021.05.002

